# Multivariate meta-analysis using individual participant data

**DOI:** 10.1002/jrsm.1129

**Published:** 2014-11-21

**Authors:** R. D. Riley, M. J. Price, D. Jackson, M. Wardle, F. Gueyffier, J. Wang, J. A. Staessen, I. R. White

**Affiliations:** a Research Institute of Primary Care and Health Sciences, Keele University, Staffordshire, ST5 5BG, UK; b School of Health and Population Sciences, Public Health Building, University of Birmingham, Edgbaston, Birmingham, B15 2TT UK; c MRC Biostatistics Unit, Cambridge, UK; d School of Mathematics, Watson Building, University of Birmingham, Edgbaston, Birmingham, B15 2TT UK; e UMR5558, CNRS and Lyon 1 Claude Bernard University, Lyon, France; f Centre for Epidemiological Studies and Clinical Trials, Ruijin Hospital, Shanghai Jiaotong University School of Medicine, Ruijin 2nd Road 197, Shanghai 200025, China; g Research Unit Hypertension and Cardiovascular Epidemiology, KU Leuven Department of Cardiovascular Sciences, University of Leuven, Leuven, Belgium; h Department of Epidemiology, Maastricht University, Maastricht, Netherlands

**Keywords:** multivariate meta-analysis, bivariate meta-analysis, multiple outcomes, correlation, individual participant data (IPD), individual patient data

## Abstract

When combining results across related studies, a multivariate meta-analysis allows the joint synthesis of correlated effect estimates from multiple outcomes. Joint synthesis can improve efficiency over separate univariate syntheses, may reduce selective outcome reporting biases, and enables joint inferences across the outcomes. A common issue is that within-study correlations needed to fit the multivariate model are unknown from published reports. However, provision of individual participant data (IPD) allows them to be calculated directly. Here, we illustrate how to use IPD to estimate within-study correlations, using a joint linear regression for multiple continuous outcomes and bootstrapping methods for binary, survival and mixed outcomes. In a meta-analysis of 10 hypertension trials, we then show how these methods enable multivariate meta-analysis to address novel clinical questions about continuous, survival and binary outcomes; treatment–covariate interactions; adjusted risk/prognostic factor effects; longitudinal data; prognostic and multiparameter models; and multiple treatment comparisons. Both frequentist and Bayesian approaches are applied, with example software code provided to derive within-study correlations and to fit the models.

## 1 Introduction

In meta-analysis, multiple summary results are required when there are multiple effects of interest, such as multiple outcomes (Berkey *et al*., 1995) or multiple time points (Dear, 1994). For example, in a meta-analysis of hypertension trials, the treatment effect on both systolic blood pressure (SBP) and diastolic blood pressure (DBP) will be of interest. In this situation, a multivariate meta-analysis model allows a joint synthesis of the multiple outcomes (Raudenbush *et al*., 1988; Becker, 2000; Van Houwelingen *et al*., 2002; Jackson *et al*., 2011). This produces a summary (pooled) effect for each outcome and accounts for any correlation in the outcome effects, which may exist both within studies and between studies. *Between-study correlation* indicates how the true effects on the outcomes are related across studies. It is caused by differences across studies in patient and study characteristics that modify the true treatment effects on the outcomes: A well-known example is the choice of threshold level in diagnostic test studies, which modifies both sensitivity and specificity (Reitsma *et al*., 2005). *Within-study correlations* indicate the association between the estimates of effect on the outcomes within a study and are caused by the same individuals contributing related data towards each outcome. In other words, within-study correlation arises because of correlated patient outcomes within a trial, and between-trial correlation arises because of correlated effects on the outcomes across studies.

Joint synthesis can improve efficiency over separate univariate syntheses (Riley *et al*., 2007a), can reduce selective outcome reporting biases (Kirkham *et al*., 2012), and allows the association between outcomes to be modelled, enabling joint inferences across outcomes and the prediction of one outcome from another, such as a surrogate outcome. However, a major stumbling block is the availability of within-study correlations, which are rarely provided in published reports. Methods and approaches have been suggested to deal with this problem (Jackson *et al*., 2011; Berkey *et al*., 1996; Riley *et al*., 2008a; Riley, 2009; Nam *et al*., 2003). However, the availability of individual participant data (IPD) from studies allows the within-study correlation to be directly calculated. Here, we illustrate how to estimate these within-study correlations using IPD and show how their availability enables multivariate meta-analysis to address clinically relevant questions about continuous, binary, survival and mixed outcomes, across wide range of applications. Some of the approaches have been suggested previously, but others are new. We focus mainly on a two-stage estimation framework (Simmonds *et al*., 2005; Riley *et al*., 2010), where the effect estimates and their variances and correlations are obtained for the outcomes in each trial separately and then synthesised in a multivariate model. The two-stage approach will be a familiar meta-analysis framework for most readers. However, one-stage approaches may be preferable for more exact modelling of the likelihood (Hamza *et al*., 2008; Trikalinos *et al*., 2013), estimating patient-level interactions (Riley *et al*., 2008b) and developing prognostic models, and so, we also briefly discuss one-stage approaches in sections 5 and 6 and the Discussion.

The article is structured as follows. Section 2 introduces a hypertension data set with multiple outcomes as motivation. Section 3 describes the multivariate meta-analysis model in detail. Subsequent sections then describe how to use IPD to produce multiple estimates in each study, along with their variances and correlations that then allow the multivariate model to be applied. Section 4 considers multiple continuous outcomes and introduces the idea of bootstrapping to estimate within-study correlations. Section 5 then considers binary, survival and mixed outcomes. Section 6 highlights some specific and novel applications of multivariate meta-analysis, and finally, section 7 concludes with discussion.

## 2 Motivating example

The methods in this article are mainly illustrated through an IPD meta-analysis of hypertension trials. Wang *et al*., 2005 investigated whether hypertension treatments lower SBP and DBP and whether they reduce the risks of a subsequent diagnosis of cardiovascular disease (CVD) or stroke. They selected randomised controlled trials that tested active anti-hypertensive drugs against placebo or no treatment, and IPD was sought from trials in the INdividual Data ANalysis of Antihypertensive intervention trials (INDANA) data set (Gueyffier *et al*., 1995) or at the Studies Coordinating Centre in Leuven (Belgium) (Liu *et al*., 1998; Staessen *et al*., 1997; Amery *et al*., 1985). Ten trials were ultimately included, providing IPD for a total of 28 581 patients (Table 1). The trials are summarised in detail elsewhere (Riley *et al*., 2008b; Wang *et al*., 2005; Riley *et al*., 2013). The treatment and control groups were well balanced in SBP and DBP in each trial at baseline (Riley *et al*., 2008b). A meta-analysis of the 10 trials is important to summarise the effect of anti-hypertension drugs on (i) reducing SBP; (ii) reducing DBP; (iii) reducing the risk of CVD; and (iv) reducing the risk of stroke. Specifically, we wish to examine the distribution of treatment effects across the trials in order to estimate the average effects of anti-hypertension drugs on these four outcomes and also to quantify the amount of between-trial variation in the outcome effects. It is also important to examine how correlated the outcome effects are, for example, to ascertain whether treatment effectiveness on SBP is correlated with treatment effect on DBP.

## 3 Multivariate meta-analysis model

Before considering the use of IPD, we begin by introducing the general approach to multivariate meta-analysis when results (in terms of effect estimates and their variances and correlations) for multiple outcomes are available from a set of primary studies.

### 3.1 General model

Let there be *K* outcomes of interest in a meta-analysis of *N* studies, and let ${\hat{\theta }}_{i}$ be a vector containing the available *K* effect estimates $\left(({\hat{\theta }}_{i1},{\hat{\theta }}_{i2},\ldots ,{\hat{\theta }}_{ik})\right)$ for the outcomes in the *i*th study (*i*=1 to *N*). The general specification of the multivariate meta-analysis model is

(1) $$\begin{array}{l} {\hat{\theta }}_{\text{i}}|\theta_{\text{i}}∼\text{N}(\theta_{\text{i},}{\text{S}}_{\text{i}})\\ \theta_{\text{i}}∼\text{N}(\text{μ},\sum ) \end{array}$$

Here, N denotes a multivariate normal distribution, **θ**_**i**_ contains the true underlying effects for the *K* outcomes for the *i*th study, **S**_**i**_ is the within-study variance–covariance matrix for the *i*th study (assumed fixed and known) containing the *K* variances of the effect estimates (in the diagonal: $S_{i1}^{2},S_{i2}^{2},\ldots \;,S_{ik}^{2}$) and their covariances (in the off-diagonal; for example, *ρ*_*Wi*(1,2)_*s*_i1_*s*_i2_ is the within-study covariance for outcomes 1 and 2, where *ρ*_*Wi*(1,2)_ is their within-study correlation), **μ** contains the *K* average effects for the outcomes, and **Σ** is the between-study variance–covariance matrix containing the *K* between-study variances of the true outcome effects (in the diagonal: $\tau_{1}^{2},\tau_{2}^{2},\ldots \;,\tau_{k}^{2}$) and their between-study covariances (in the off-diagonal; e.g. the between-study covariance for outcomes 1 and 2 is *ρ*_*B*(1,2)_*τ*_1_*τ*_2_, where *ρ*_*B*(1,2)_ is their between-study correlation). The number of rows in each vector is equal to the number of outcomes.

If some studies have missing outcome effect estimates (i.e. one or more of the entries of ${\hat{\theta }}_{i1},{\hat{\theta }}_{i2},\ldots \;,{\hat{\theta }}_{iK}$ are missing) then, assuming that these are missing at random and for computational convenience, such studies can be incorporated by allocating them arbitrary values (e.g. set ${\hat{\theta }}_{i1}=0$ if missing) with very large within-study variances (e.g. set $S_{i1}^{2}$ = 1 000 000 if ${\hat{\theta }}_{i1}$ is missing) and within-study correlations of 0. This replaces missing outcome effects with estimates that have negligible weight and information during estimation.

We take **Σ** to be unstructured in this article, but simplifications are possible; for example, all between-study correlations, or all between-study variances, could be assumed to be the same. Between-study correlations may be increasingly difficult to estimate as the number of outcomes increases, with frequentist estimates often at +1 or −1 when the number of studies is few, or the between-study heterogeneity is small relative to the within-study variability (Riley *et al*., 2007b). Furthermore, in the presence of missing outcomes in some studies, there must be enough estimated effects, and combinations of these within studies, to make **Σ** identifiable. This is true for our applications throughout the article. A multivariate fixed effect model is obtained when **Σ** is **0**, and model (1) is equivalent to two independent univariate random-effect meta-analyses when all within and between-study correlations are assumed zero.

### 3.2 Estimation

Estimation of model (1) is needed to obtain estimates of **μ** and **Σ**. Estimation can be achieved by a variety of options (Jackson *et al*., 2011), including restricted maximum likelihood (REML), method of moments (MM) approaches (Jackson *et al*., 2010; Jackson *et al*., 2013; Chen *et al*., 2012), the U statistic (Ma and Mazumdar, 2011), and a Bayesian approach (Nam *et al*., 2003; Wei and Higgins, 2013a; Bujkiewicz *et al*., 2013). In this article, we present results using REML and Bayesian estimation. REML does not by default account for the uncertainty about $\hat{\Sigma }$ in the estimation of **μ**, but confidence intervals (CIs) can be inflated to reflect the uncertainty (White, 2009; Jackson and Riley, 2014). After model estimation, multivariate extensions to *I*^2^ can be calculated (Jackson *et al*., 2012), for each outcome separately and across both outcomes jointly. They express the fraction of variation in the meta-analysis that is because of between-study heterogeneity rather than within-study sampling error.

For the Bayesian approach, prior distributions are necessary for **μ** and **Σ**. We give a vague separate normal prior distribution for each component of **μ**, with a mean of zero and a large variance of 1 000 000. The prior distribution is harder to specify for **Σ**, especially when there are three or more outcomes. The conjugate prior distribution for **Σ** is an inverse Wishart distribution, but this is known to be potentially informative, and separation of the components of **Σ** is preferred to allow realistically vague prior distributions on each term (Web Appendix 1) (Wei and Higgins, 2013a; Barnard *et al*., 2000). **Σ** is first separated into variance and correlation matrices by **Σ** = **V**^**1/2**^**RV**^**1/2**^, where **V**^**1/2**^ is a diagonal matrix with between-study standard deviations as elements and **R** is the *k* × *k* matrix of between-study correlations (where *k* is the total number of outcomes). Then, **R** is re-parameterised to enforce positive-definite constraints. Cholesky decomposition or spherical decomposition can achieve this, and we refer the reader to Wei and Higgins who describe this in full (Wei and Higgins, 2013a). Our WinBUGS code for a four outcome multivariate meta-analysis using either decomposition is given in Web Appendix 1. A product-normal specification of **Σ** is also possible (Spiegelhalter *et al*., 2000; Bujkiewicz *et al*., 2013).

In the bivariate setting, an example set of prior distributions is

$$\begin{array}{ll} \;\mu_{1}∼\text{N}(0,1000000) & \mu_{2}∼\text{N}(0,1000000)\\ \;\tau_{1}∼\text{N}(0,1)\text{I}(0) & \;\tau_{2}∼\text{N}(0,1)\text{I}(0)\\ \rho_{B(1,2)}∼\;\text{Uniform}(-1,1) & \end{array}$$

where *ρ*_*B*(1,2)_ is the between-study correlation of *θ*_*i*1_ and *θ*_*i*2_. WinBUGS code for the bivariate setting is given in Web Appendix 2. In situations where vague prior distributions are required, we suggest that a number of prior distributions are examined, especially when the number of studies is small, as the choice of prior distributions for *τ*_1_, *τ*_2_ and *ρ*_*B*(1,2)_ may affect the posterior results (Lambert *et al*., 2005). Of course, external evidence regarding *τ*_1_, *τ*_2_ and *ρ*_*B*(1,2)_ can be incorporated in the prior distributions where available. For example, if *ρ*_*B*(1,2)_ is considered positive, then its prior distribution could be chosen to allow only positive values.

### 3.3 Advantages over univariate meta-analysis

Model (1) is particularly advantageous over separate univariate meta-analyses of each outcome when some outcomes are systematically missing (i.e. only one of the outcomes is available) in some of the studies (Riley *et al*., 2007a) or when inferences are to be made *jointly* about the average effects of two or more outcomes (e.g. *μ*_1_ and *μ*_2_) (Thompson *et al*., 2005) or *jointly* about the predicted outcome effects (e.g. *θ*_*i*1_ and *θ*_*i*2_) in a new population (Buyse *et al*., 2000). Such predictions are perhaps most suited to the Bayesian framework, in particular to propagate the uncertainty in $\hat{\Sigma }$ and allow direct probability statements about the magnitude of effects for both outcomes. Web Appendices 2 and 3 gives examples of calculating such probability statements from a Bayesian analysis.

## 4 Multivariate IPD meta-analysis of continuous outcomes

Here onwards, we concentrate on facilitating the multivariate model (1) by using IPD to obtain the necessary effect estimates, and their within-study variances and covariances, from each study. We begin by considering multiple continuous outcomes, such as SBP and DBP, when given IPD from *N* randomised trials. In the first stage, each of these studies is analysed separately, and then in the second stage, model (1) is applied, as now described.

### 4.1 First stage

Assume that there is a treatment group (T) and a control group (C) in each trial and that two continuous outcomes (*k* = 1 or 2) are of interest. At baseline (i.e. before randomisation), the *j*th patient in the *i*th trial provides their SBP and DBP values, which we denote by *y*_B*ijk*_, where B indicates baseline and *k* = 1 for SBP and *k* = 2 for DBP. Also, each patient provides their final SBP and DBP values after treatment, which we denote by *y*_F*ijk*_ (where F indicates final). Let *x*_*ij*_ be 0/1 for patients in the control/treatment group respectively. When such data are available, the first step of a two-step IPD multivariate meta-analysis must estimate the treatment effects for each outcome in each trial.

#### 4.1.1 Option 1: modelling outcomes separately within each trial

The most appropriate linear regression model to use in this situation is analysis of covariance (ANCOVA) (Riley *et al*., 2013), where the outcome follow-up value is the response variable and the treatment effect is estimated adjusted for the baseline value. For each outcome and each trial separately, the ANCOVA model to be fitted is

(2) $$\begin{array}{l} y_{Fijk}=\Phi_{ik}+\beta_{ik}y_{Bijk}+\theta_{ik}x_{ij}+e_{ijk}\\ \;e_{ijk}∼N(0,\sigma_{ik}^{2}) \end{array}$$

In this model, *ɸ*_*ik*_ is the fixed trial effect for outcome *k*, *β*_*ik*_ denotes the mean change in *y*_F*ijk*_ for a one-unit increase in *y*_*Bijk*_, *θ*_*ik*_ is the underlying treatment effect for outcome *k* in trial *i*, and $\sigma_{ik}^{2}$ is the residual variance of outcome *k* in trial *i* after accounting for treatment and baseline values. The model is simply a linear regression and so can easily be estimated in standard statistical software. From this, the treatment effect estimate,${\hat{\theta }}_{ik}$, and its variance, $s_{ik}^{2}$, are obtained for each outcome in each trial. If baseline values are unavailable, then model (2) excluding the baseline value as a covariate will still give unbiased ${\hat{\theta }}_{ik}$ when patients are randomised to the treatment groups, although ${\hat{\theta }}_{ik}$ will be less efficient.

#### 4.1.2 Option 2: modelling outcomes jointly within each trial

Model (2) can be applied to each outcome separately, but for continuous outcomes, one can simultaneously fit model (2) for both outcomes as follows:

(3) $$\begin{array}{l} y_{Fijk}=\varphi_{ik}+\beta_{ik}y_{Bijk}+\theta_{ik}x_{ij}+e_{ijk}\\ \;e_{ijk}∼N(0,\sigma_{ik}^{2})\\ \mathrm{cov}(e_{ij1},e_{ij2})=\sigma_{i12} \end{array}$$

In this framework, each patient now contributes two follow-up responses and two baseline values (one for each outcome) to a single model containing both outcomes jointly (Riley *et al*., 2008b). It is thus similar to a repeated measure model, and the correlation in patient outcome responses is accounted for by the covariance term, *σ*_*i*12_. For estimation purposes, it is helpful to re-write model (3) with dummy variables as shown in Web Appendix 3, with associated SAS code. When each patient provides both outcomes, then model (3) will give approximately the same treatment effect estimates and standard errors as obtained from fitting model (2) to each outcome separately. However, model (3) can also include patients with one of the outcomes missing and, under a missing at random assumption, will utilise the patient-level correlation to ‘borrow strength’ across outcomes. In situations with a large proportion of incomplete outcome data (i.e. only one outcome observed), this extra information may lead to different treatment effect estimates with lower variances than from model (2). Such a scenario is unlikely in well-designed prospective studies such as randomised trials.

#### 4.1.3 Estimating within-study correlations directly or via bootstrapping

An important part of a multivariate meta-analysis is to estimate and utilise the within-study correlations between the outcome effect estimates. For example, consider two outcomes (such as SBP and DBP), and let the within-study correlation of ${\hat{\theta }}_{i1}$ and ${\hat{\theta }}_{i2}$ in trial *i* be denoted by *ρ*_*Wi*(1,2)_, where

(4) $$\rho_{Wi(1,2)}=\frac{\text{cov}\left({\hat{\theta }}_{i1},{\hat{\theta }}_{i2}\right)}{s_{i1}s_{i2}}=\frac{s_{i(1,2)}}{s_{i1}s_{i2}}$$

Model (3) models the covariance between the patient outcome responses, and this naturally induces a within-study correlation (*ρ*_*Wi*(1,2)_) between the estimated treatment effects, ${\hat{\theta }}_{i1}$ and ${\hat{\theta }}_{i2}$. After estimation of model (3), *ρ*_*Wi*(1,2)_ is therefore calculable directly using the variances, $s_{i1}^{2}$, and $s_{i2}^{2}$ the covariance, *s*_*i*__(1,2)_, available from the inverse of Fisher’s information matrix. Statistical software such as SAS (Web Appendix 3) provides these results upon request.

Model (2), however, does not model patient-level correlation and analyses each outcome separately; therefore, the within-study correlations are not estimated. However, they can be estimated via non-parametric bootstrapping. This approach randomly selects one patient with replacement, then randomly selects a second patient with replacement, and repeats until the same sample size is obtained as in the trial. This process is repeated *b* times so that *b* bootstrap samples are obtained. Then, in each of the bootstrap samples, model (2) is fitted to outcome 1 (e.g. SBP) to obtain ${\hat{\theta }}_{i1}$ and then to outcome 2 (e.g. DBP) to obtain ${\hat{\theta }}_{i2}$. This produces *b* values of ${\hat{\theta }}_{i1}$ and ${\hat{\theta }}_{i2}$, and their observed correlation can be calculated, which gives *ρ*_*Wi*(1,2)_. When *b* is large, the *ρ*_*Wi*(1,2)_ estimated from bootstrapping should be very similar to the *ρ*_*Wi*(1,2)_ estimated directly from model (3) (this is demonstrated in section 4.3 and Table 1).

### 4.2 Second stage

The first stage therefore provides treatment effect estimates $\left({\hat{\theta }}_{ik}\right)$ and their variances $\left(s_{ik}^{2}\right)$, for each outcome in each trial, and their within-study correlations (e.g. *ρ*_*Wi*(1,2)_). Multivariate model (1) can now be implemented. For example, if there are two outcomes, then model (1) becomes a bivariate random-effect meta-analysis, which written in full is (Van Houwelingen *et al*., 2002; Jackson *et al*., 2011)

(5) $$\begin{array}{cc} \left(\begin{array}{c} {\hat{\theta }}_{i1}\\ {\hat{\theta }}_{i2} \end{array}\right)∼N\left(\left(\begin{array}{c} \theta_{i1}\\ \theta_{i2} \end{array}\right),{\text{S}}_{i}\right) & \;{\text{S}}_{i}=\left(\begin{array}{cc} s_{i1}^{2} & \rho_{Wi(1,2)}s_{i1}s_{i2}\\ \rho_{Wi(1,2)}s_{i1}s_{i2} & s_{i2}^{2} \end{array}\right)\\ \left(\begin{array}{c} \theta_{i1}\\ \theta_{i2} \end{array}\right)∼N\left(\left(\begin{array}{c} \mu_{1}\\ \mu_{2} \end{array}\right),\Sigma \right) & \Sigma =\left(\begin{array}{cc} \tau_{1}^{2} & \rho_{B(1,2)}\tau_{1}\tau_{2}\\ \rho_{B(1,2)}\tau_{1}\tau_{2} & \tau_{2}^{2} \end{array}\right) \end{array}$$

### 4.3 Application to the hypertension data

#### 4.3.1 Within-study correlations

**Figure 1.**
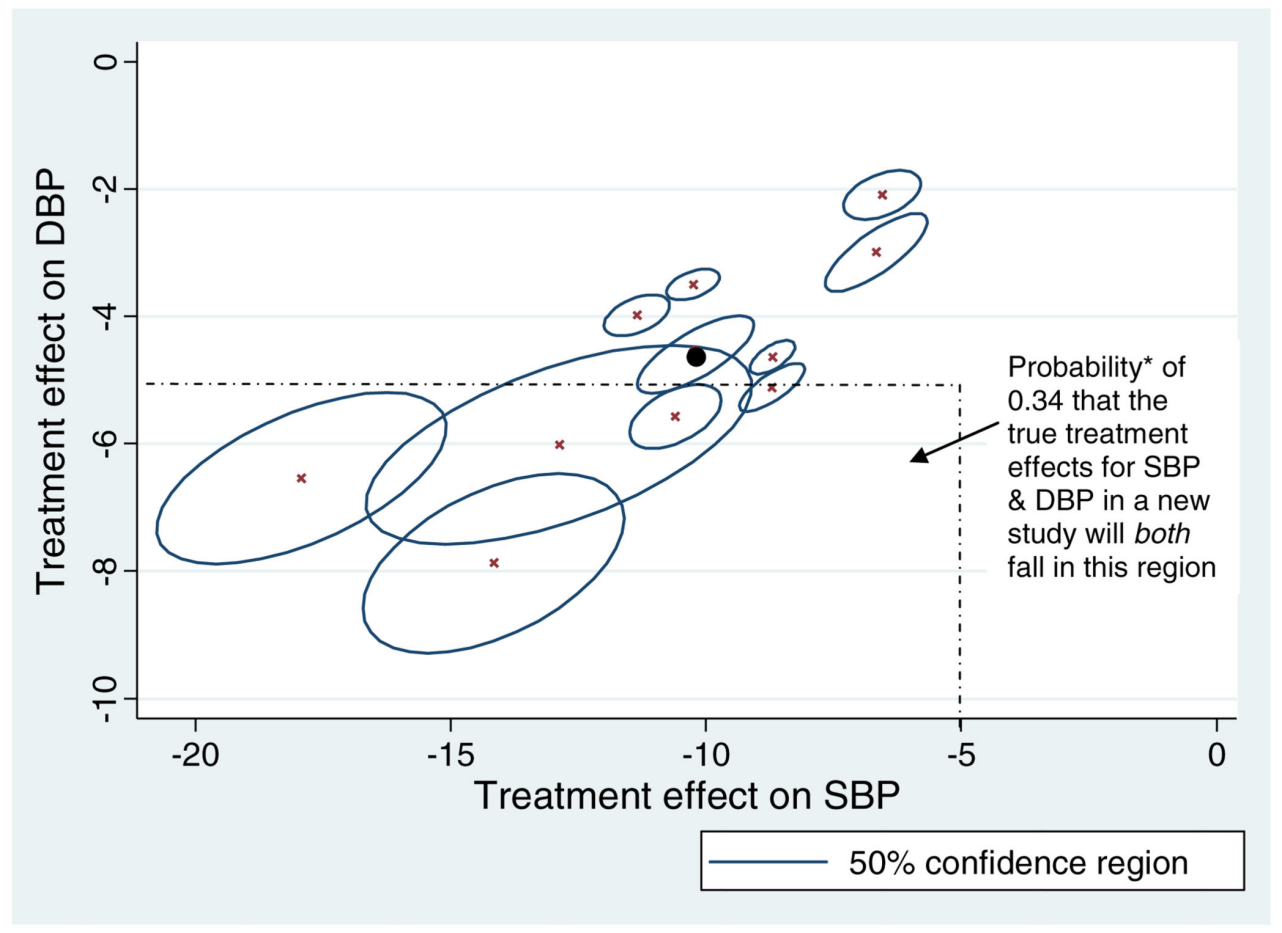
Relationship between the treatment effect estimates on systolic blood pressure (SBP) and diastolic blood pressure (DBP), within and across trials. The crosses indicate a pair of estimates from one trial, and the angle of the confidence ellipse around each estimate indicates the within-study correlation. The solid circle denotes the pair of summary estimates from the meta-analysis, and the circle around it denotes its confidence ellipse. 50% (rather than 95%) confidence ellipses are given for cosmetic reasons, as otherwise the regions are large and overlap considerably. * estimated from a Bayesian bivariate meta-analysis model, with prior distributions as specified in section 3.2. This is the proportion of the joint posterior distribution for the underlying treatment effects of these two outcomes that falls within this region.

The joint ANCOVA model (3) was applied to each of the hypertension trials separately, to estimate the treatment effect on SBP and DBP after adjusting for baseline values. The treatment effect estimates and their variances for SBP and DBP are shown in Table 1, together with their within-study correlations that are all positive and quite high (ranging from 0.45 to 0.79). They are shown visually through the direction of the confidence ellipse for the effects in each trial (Figure 1). Therefore, there is a strong association between the treatment effect estimates for SBP and DBP, a consequence of the high correlation between SBP and DBP values in individuals, and this justifies why consideration of correlation is important. The within-study correlations estimated via model (2) with bootstrapping were very similar (Table 1).

#### 4.3.2 Between-study correlation

The trial treatment effect estimates were combined in a bivariate meta-analysis model (5), using within-study correlations estimated via bootstrapping. The frequentist results after REML estimation are shown in Table 2. Bayesian analyses led to similar conclusions and are thus not presented in full here; however, the WinBUGS code to replicate the analysis is shown in Web Appendix 2. REML estimates a high positive between-study correlation of 0.78, indicating that trials with a higher than average true treatment effect on SBP also have a higher than average true treatment effect on DBP. This can be seen visually in Figure 1 by looking at the relationship across trials. Thus, if for some reason the treatment effect on DBP was not observed in a trial, one might use the high between-study correlation to predict the effect on DBP from the observed effect on SBP.

#### 4.3.3 Summary treatment effects

The REML summary treatment effect estimates indicate that, on average, hypertension treatment reduces both SBP (${\hat{\mu }}_{1}=-10.21$, 95% CI: −12.11 to −8.30) and DBP (${\hat{\mu }}_{2}=-4.59$, 95% CI: −5.61 to −3.57) by more than placebo. The Bayesian approach obtains similar estimates and gives a posterior probability of almost 1 that *μ*_1_ < 0 and a probability almost 1 that *μ*_2_ < 0, providing very strong evidence that treatment is effective at reducing each outcome. One can also make joint inferences. For example, there is a probability of 0.12 that treatment will reduce *both* SBP and DBP by at least 5 mmHg on average, which illustrates the use of a more stringent minimum reduction for clinical acceptability. One might also be interested in the average treatment effect on pulse pressure (SBP – DBP), which is ${\hat{\mu }}_{1}-{\hat{\mu }}_{2}=5.61$ (95% CI: −6.94 to −4.29) using the REML estimates. We note that ignoring the correlation between ${\hat{\mu }}_{1}$ and ${\hat{\mu }}_{2}$ produces a more conservative CI of −7.73 to −3.42.

#### 4.3.4 Predicted treatment effects in a new population

There is heterogeneity in treatment effect across trials (${\hat{\tau }}_{1}=2.71,{\hat{\tau }}_{2}=1.48$ from REML estimation), potentially caused by differences in patient-level and study-level characteristics. Given the heterogeneity, clinicians might not be interested in the average treatment effect across trials but rather the predicted treatment effect (*θ*_*new*1_ and *θ*_*new*2_) when it is implemented in a new population. Interest then lies in the bivariate distribution of *θ*_*new*1_ and *θ*_*new*2_ for a new study, that is, the joint distribution of true treatment effects across new studies for the two outcomes. The Bayesian approach gives a 95% prediction interval of −14.11 to −5.82 for the treatment effect on SBP and −7.28 to −1.75 for the treatment effect on DBP. These intervals account for the uncertainty in ${\hat{\mu }}_{1},{\hat{\mu }}_{2}$ and $\hat{\Sigma }$. There is a probability of 0.99 that treatment will reduce both SBP and DBP more than placebo in a new population. Further, there is a probability of 0.34 that the treatment will reduce both SBP and DBP by at least 5 mmHg more than placebo in a new population. This is the probability that the two predicted effects will fall in the marked area of Figure 1.

## 5 Multivariate IPD meta-analysis for binary, survival and mixed outcomes

We now extend the two-stage framework of section 4 to consider binary, survival, and mixed outcomes.

### 5.1 Binary outcomes

Often, researchers are interested in multiple binary outcomes, and these may also be related to one another, especially if they are nested or mutually exclusive. Nested outcomes occur when one outcome is a subset of the other, such as *unplanned caesarean section* and *any adverse maternal outcome* in pregnancy. Mutually exclusive outcomes occur when patients with one outcome cannot experience the other, for example, *delivery by caesarean section* and *instrumental delivery other than caesarean section*. In each trial, within IPD, those nested or mutually exclusive binary outcomes can be identified, and the number of events obtained for each (although, such information may also be available in study publications, for example, in contingency tables). Treatment effect estimates such as of log odds ratios (ORs) or log relative risks can then be estimated for each outcome in each study, along with their within-study variances. Their within-study correlations can be estimated by the formulae provided by Trikalinos and Olkin, 2008 for mutually exclusive outcomes and Wei and Higgins, 2013b for nested outcomes. Then, multivariate meta-analysis model (1) can be applied.

Multivariate meta-analysis model (1) can also be used to jointly synthesise effects for related binary outcomes that are neither nested nor mutually exclusive, such as *pain-free walk by 1 month* and *reduction in swelling by 1 month* after a knee operation. Wei and Higgins, 2013b provide formulae for calculating within-study correlations for treatment effects for these outcomes, which requires an estimate of the patient-level correlation of outcome responses, which could easily be derived with the IPD.

It is also straightforward to implement the bootstrap procedure described in section 4.1.3 to estimate their within-study correlation. For example, one can fit to each trial and each outcome separately a logistic regression model:

(6) $$\text{In}\;\left(\frac{p_{ijk}}{1-p_{ijk}}\right)=\alpha_{ik}+\theta_{ik}x_{ij}$$

where *p*_*ijk*_ is the probability of patient *j* in study *i* experiencing outcome *k*, and α_*ik*_ is the log odds of outcome *k* in study *i* for the control group, *x*_*ij*_ is 1 for those in the treatment group and 0 for control group, and *θ*_*ik*_ denotes the log OR (i.e. the treatment effect) in the *i*th study for outcome *k*. The model can be estimated by maximum likelihood, to give the treatment effect estimate ${\hat{\theta }}_{ik}$ and its variance $s_{ik}^{2}$ for each outcome in each trial. Baseline covariates might also be included alongside *x*_*ij*_ in order to increase power or adjust for baseline confounding. To estimate the within-study correlations, the bootstrap is then used, where now, logistic regression model (6) is applied to each of the *b* bootstrap samples, producing *b* pairs of log OR estimates for each pair of outcomes and thereby enabling their correlation to be estimated.

Within-study correlations do not need to be derived if, rather than a two-stage framework, a one-stage multivariate meta-analysis is rather performed. For example, for nested or mutually exclusive outcomes, Trikalinos *et al*., 2014 show how multinomial distributions can be used to model the number of events within studies whilst incorporating random effects to allow for between-study heterogeneity and correlation in the true log ORs. The use of discrete within-study likelihoods in this way avoids the (potentially inappropriate) large sample normality assumption of the log OR estimates in each study and avoids the need for continuity correlations (Stijnen *et al*., 2010). However, in our experience, such one-stage meta-analyses can sometimes lead to convergence problems (Riley *et al*., 2007b), especially when trying to accommodating studies with missing outcomes, and thus, the two-stage approach may often be more practical.

#### 5.1.1 Application to the hypertension data

Clinicians typically define a normal SBP as ≤120 mmHg and a normal DBP as ≤80 mmHg. We now consider these as two binary outcomes and evaluate whether hypertensive treatment improves the odds of having a normal SBP and DBP by the end of the trial. Model (6) was applied to each outcome in each study, to obtain the treatment effect estimates and their variances (data shown in Web Appendix 4). Bootstrapping estimated positive within-study correlations, ranging from +0.05 to +0.48. Multivariate meta-analysis model (1) was then applied using REML, and the summary results show that, compared with control, hypertension treatment improves the odds of having a normal SBP and DBP (Table 2). Interestingly, the summary result and 95% CI for DBP are similar whether univariate or multivariate meta-analysis is applied (OR = 2.34, 95% CI: 2.01 to 2.72). However, for SBP, the summary result has a wider CI in the multivariate (OR = 2.45, 95% CI: 1.96 to 3.06) compared with univariate (OR = 2.46, 95% CI: 2.19 to 2.76) meta-analysis, as a result of a larger estimate of between-study standard deviation (0.12 vs 0.043). This highlights how multivariate meta-analysis also borrows strength across outcomes for the estimates of heterogeneity. The treatment effect estimates for SBP have large variances in some trials, as a result of only a few patients obtaining a normal SBP. This imbalance in SBP variances across studies improves the opportunity to borrow strength (Riley *et al*., 2007a) from the treatment effect estimates for DBP, which have consistently smaller variances (Web Appendix 4), via the within-study correlations and between-study correlation of +0.35.

### 5.2 Survival outcomes

Often, researchers are interested in a treatment effect on multiple time-to-event (survival) outcomes, for example, the time to disease recurrence and the time to death. These may also be correlated, and thus, multivariate meta-analysis is again appealing. For simplicity in this paper, we assume that a Cox proportional hazard model is appropriate for all time-to-event outcomes. However, we recognise that with IPD, more sophisticated multivariate survival models (Wassell and Moeschberger, 1993; Hougaard, 2000; Burzykowski *et al*., 2001; Michiels *et al*., 2009), multistate modelling (Putter *et al*., 2007; Price *et al*., 2011), and one-stage meta-analyses (Crowther *et al*., 2012; Rondeau *et al*., 2011) are possible, and outcomes with competing risks can be synthesised using more sophisticated analyses (Ades *et al*., 2010). We do not have space to consider such topics in sufficient detail here and so focus our attention on facilitating multivariate meta-analysis using standard approaches. Therefore, in the event of death, other outcomes that have not occurred are censored at the death time.

Given IPD, one can fit to each trial and each outcome separately a Cox model (Cox, 1972)

(7) $$h_{ijk}\left(t\right)=h_{0ik}\left(t\right)\text{exp}\left(\theta_{ik}x_{ij}\right)$$

where *h*_*ijk*_(*t*) is the hazard function over time *t* for having an outcome *k* event for the *j*th individual in the *i*th trial. Each trial has its own baseline hazard (*h*_0*ik*_(*t*)), which denotes the hazard of an event in the control group for outcome *k*, and the parameter *θ*_*ik*_ denotes the log hazard ratio (i.e. the treatment effect) in the *i*th study for outcome *k*. The model can be estimated by numerical maximisation of the partial likelihood (Cox, 1972), to give the treatment effect estimate ${\hat{\theta }}_{ik}$ and its variance $s_{ik}^{2}$ for each outcome in each trial. Baseline covariates might also be included alongside *x*_*ij*_ in order to increase power. A method for handling tied failure times in the calculation of the log partial likelihood may also be required; here, we use the Breslow method (Breslow, 1975).

Applying model (7) to each outcome separately does not provide the within-study correlations between the multiple ${\hat{\theta }}_{ik}\text{s}$ of interest. However, as described for the logistic regression models for binary outcomes, they can be estimated using the bootstrapping procedure described in section 4.1.3. This is also highlighted elsewhere (Bujkiewicz *et al*., 2013). Once estimated, the multivariate meta-analysis model (1) can then be fitted.

#### 5.2.1 Application to the hypertension data

Consider the two outcomes of CVD and stroke. Model (7) was used in each trial to estimate the treatment effect on CVD incidence rate and then again to estimate the treatment effect on stroke incidence rate. Bootstrapping was used to estimate the within-study correlations, and these are all positive and often high (ranging from 0.10 to 0.78) (Table 1). This suggests an underlying strong positive association at the individual level between reduction in stroke incidence and reduction in CVD incidence from using the treatment. This is expected, as stroke is one of the main reasons for a diagnosis of CVD (others include angina, heart attack, and heart failure).

Estimation of bivariate meta-analysis model (5) using REML gives a between-study correlation of +1 (Table 2); however, this is poorly estimated and has very little impact on the summary results because both between-study variances are estimated to be almost zero. Thus, the results are similar to those from a bivariate fixed effect meta-analysis, and the lack of heterogeneity suggests that the effect of treatment may be consistent across populations. The summary hazard ratios less than 1 indicate that hypertension treatment is effective at reducing both stroke and CVD risk (Table 2). In comparison to univariate meta-analyses, the large within-study correlations allow borrowing of strength across outcomes, and this produces slightly narrower CIs as a result of smaller standard errors. For example, for stroke, the standard error of the summary log hazard ratio is reduced from 0.074 in the univariate analysis to 0.070 in the bivariate analysis, a reduction of about 5%. Joint inferences can also be made accounting for the correlation. For example, following a bivariate Bayesian analysis, the estimated probability that hypertension treatment will reduce the hazard of stroke and CVD by at least 20% in a new study is 0.52. If one wrongly ignores the correlation between outcomes, the estimated probability is lower at 0.47. In situations where different treatment options are being compared (e.g. when multiple treatments exist for the same disease or when resource limits the number of treatments that can be purchased by a particular health care body), such discrepancies in the estimated probability of success may impact upon the priority ranking of each treatment (refer to Network meta-analysis, section 6.5).

### 5.3 Mixed outcomes

So far, we have considered multivariate meta-analysis for multiple outcomes of the same type. However, multivariate meta-analysis can also be applied for mixed outcomes, For example, in the hypertension example, the treatment effects on the continuous outcomes of SBP and DBP can be considered jointly with the treatment effects on the survival outcomes of stroke and CVD. The availability of IPD allows within-study correlations between these mixed outcomes to be estimated, with bootstrapping again a convenient approach. Here, in the *b* bootstrap samples for each study, different types of analyses can be fitted to each outcome: for example, an ANCOVA for SBP, an ANCOVA for DBP, a Cox regression for CVD, and a Cox regression for stroke. These provide *b* sets of treatment effect estimates, and their correlations give the within-study correlations. Example STATA code is provided in Web Appendix 5 to do this.

#### 5.3.1 Application to the hypertension dataset

The REML results for the multivariate meta-analysis of all four outcomes (SBP, DBP, stroke and CVD) show that hypertension treatment is effective for all four outcomes on average, with the summary effect for SBP and DBP <0 and the summary hazard ratio for stroke and CVD <1 (Table 2; NB the summary hazard ratios are the exponential of the summary log_e_ hazard ratio estimates from the multivariate model). Bayesian estimates were very similar (available on request). Interestingly, after accounting for the between-study correlations with SBP and DBP, the multivariate model (1) provides larger estimates of between-study heterogeneity for both stroke and CVD (compared with the bivariate results in section 4.4.1). This is because of the borrowing of strength across outcomes for the average effects *and* the heterogeneity, as mentioned previously.

**Figure 2.**
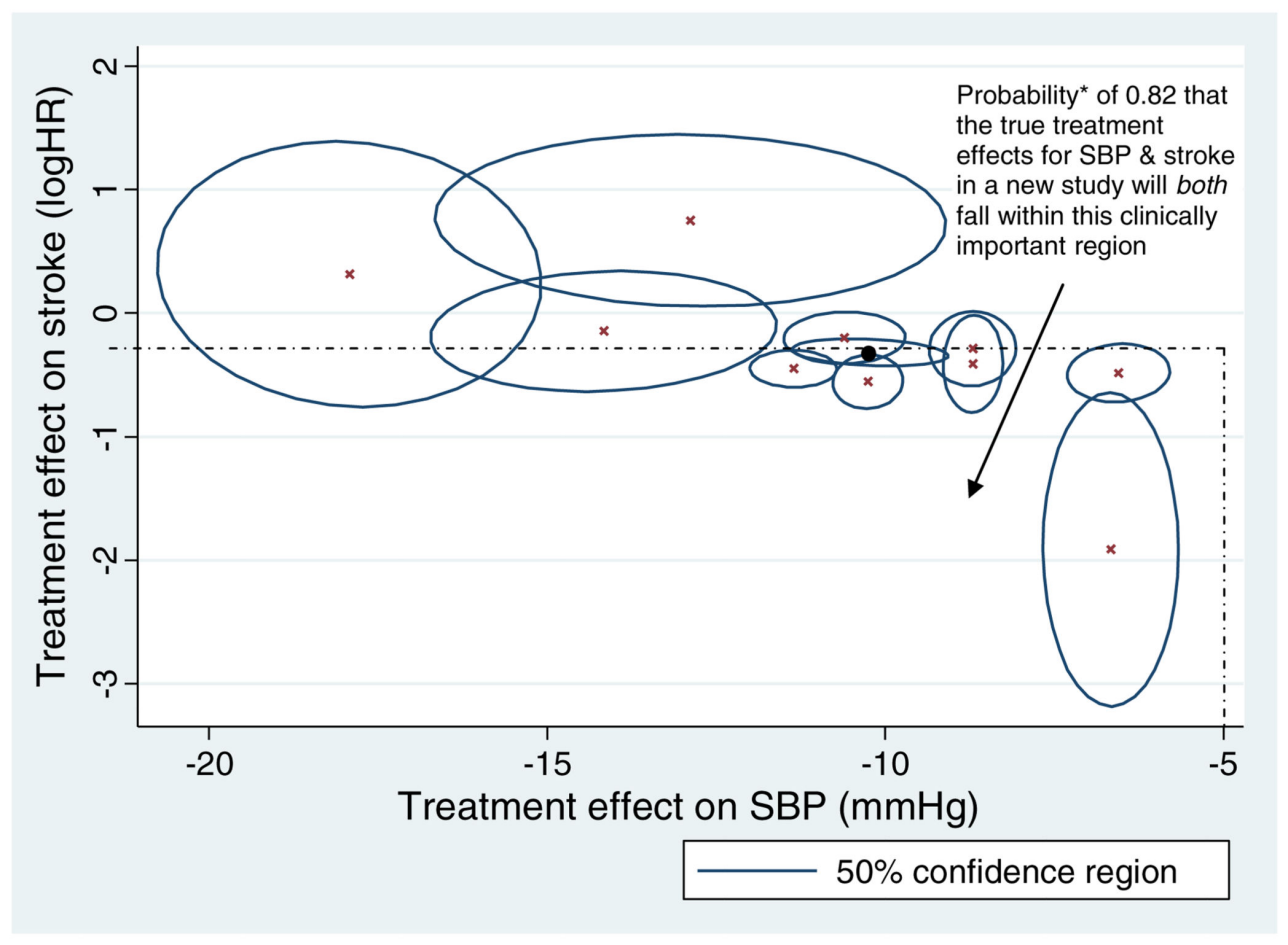
Relationship between the treatment effect estimates on systolic blood pressure (SBP) and stroke, within and across trials. The crosses indicate a pair of estimates from one trial, and the angle of the confidence ellipse around each estimate indicates the within-study correlation. The solid circle denotes the pair of summary estimates from the meta-analysis, and the circle around it denotes its confidence ellipse. 50% (rather than 95%) confidence ellipses are given for cosmetic reasons, as otherwise the regions are large and overlap considerably. * estimated from a Bayesian four-outcome multivariate meta-analysis model, with prior distributions as specified in section 3.2 and Web Appendix 1. This is the proportion of the joint posterior distribution for the underlying treatment effects of these two outcomes that falls within this region.

The multivariate approach allows joint probability statements across the mixed outcomes. For example, the Bayesian multivariate meta-analysis estimates that, if applied to a new population, the probability that hypertension treatment will reduce SBP by at least 5 mmHg *and* reduce the hazard of stroke by at least 20% is 0.82. This is illustrated by the probability of the effects falling in the shaded area of Figure 2.

## 6 Special applications of multivariate meta-analysis

So far, we have considered multivariate meta-analysis to jointly estimate treatment effects on several outcomes. We now consider some different applications where IPD multivariate meta-analysis might be useful.

### 6.1 Treatment–covariate interactions

Clinicians often want to know how treatment effect is modified by clinical and patient characteristics, such as age, sex or biomarker levels. It may therefore be important to extend the models in previous sections to include patient-level covariates and estimate treatment–covariate interactions.

Assume that IPD are available from all trials and there is just one outcome of interest. Let *z*_*ij*_ be a patient-level covariate (e.g. the age of patient *j* in trial *i*), which is observed for all patients in each trial. Simmonds and Higgins, 2007 suggest a two-step method for estimating the treatment–covariate interaction, and we extend their approach here, focusing on one continuous outcome. As in section 4, the first stage involves fitting a model to the IPD from each trial separately, akin to model (2) but here for just one outcome and with an interaction term (Riley *et al*., 2008b; Higgins *et al*., 2001):

(8) $$\begin{array}{l} y_{Fij}=\varphi_{i}+\beta_{i}y_{Bij}+\theta_{i}x_{ij}+\zeta_{i}z_{ij}+\gamma_{Wi}x_{ij}z_{ij}+\varepsilon_{ij}\\ \;\varepsilon_{ij}∼N\left(0,\sigma_{i}^{2}\right) \end{array}$$

The parameters are as explained under model (2) with now *θ*_*i*_ the treatment effect for those patients with *z*_*ij*_ = 0; *ζ*_*i*_ is the effect of a one-unit increase of covariate *z*_*ij*_ on blood pressure outcome (*y*_*Fij*_); and *γ*_*wi*_ is the change in treatment effect for a one-unit increase in *z*_*ij*_. In other words, *γ*_*wi*_ is the within-trial treatment–covariate interaction, using language consistent with articles on interaction estimates elsewhere (Riley *et al*., 2008b; Riley and Steyerberg, 2010). When fitted to each trial’s IPD, the model produces an interaction estimate, ${\hat{\gamma }}_{Wi}$, and its variance, $\text{V}\left({\hat{\gamma }}_{Wi}\right)$, for each trial. These can be synthesised across trials in a standard univariate meta-analysis model (Riley *et al*., 2008b; Simmonds and Higgins, 2007), such as a random-effect model:

(9) $$\begin{array}{l} {\hat{\gamma }}_{Wi}∼N\left(\gamma_{Wi},\text{V}\left({\hat{\gamma }}_{Wi}\right)\right)\\ \gamma_{Wi}∼N\left(\mu_{\gamma_{w}},\tau_{\gamma_{w}}^{2}\right) \end{array}$$

$\text{V}\left({\hat{\gamma }}_{Wi}\right)$ is assumed known, and the model provides a summary estimate of the within-trial treatment–covariate interaction, *μ*_*γw*^′^_ and the between-trial heterogeneity in the interaction $\left(\tau_{\gamma w}^{2}\right)$.

If the covariate *z*_*ij*_ is binary (let us say, 1 for males and 0 for females), then *μ*_*γw*_ gives the mean change in treatment effect for males compared with females. However, along with this contrast, it may also be informative to know the summary treatment effects for males and females separately. Therefore, as an alternative to model (9), one might fit the following bivariate meta-analysis to the treatment effect estimates for females $\left({\hat{\theta }}_{i}\right)$ and males $\left({\hat{\theta }}_{i}+{\hat{\gamma }}_{Wi}\right)$ and their variances $\text{V}\left({\hat{\theta }}_{i}\right)$ and $\text{V}\left({\hat{\theta }}_{i}+{\hat{\gamma }}_{wi}\right)$:

(10) $$\begin{array}{l} \left(\begin{array}{c} {\hat{\theta }}_{i}+{\hat{\gamma }}_{wi}\\ {\hat{\theta }}_{i} \end{array}\right)\sim N\left(\left(\begin{array}{c} \mu_{\delta }\\ \mu_{\theta } \end{array}\right),{\text{S}}_{i}+\Omega \right)\\ \;\text{S}i=\left(\begin{array}{cc} \text{V}\left({\hat{\theta }}_{i}+{\hat{\gamma }}_{wi}\right) & \rho_{wi(1,2)}\sqrt{\text{V}\left({\hat{\theta }}_{i}+{\hat{\gamma }}_{wi}\right)\text{V}\left({\hat{\theta }}_{i}\right)}\\ \rho_{wi(1,2)}\sqrt{\text{V}\left({\hat{\theta }}_{i}+{\hat{\gamma }}_{wi}\right)\text{V}\left({\hat{\theta }}_{i}\right)} & \text{V}\left({\hat{\theta }}_{i}\right) \end{array}\right)\\ \;\Omega =\left(\begin{array}{cc} \tau_{\sigma }^{2} & \rho_{B(1,2)}\tau_{\delta }\tau_{\theta }\\ \rho_{B(1,2)}\tau_{\delta }\tau_{\theta } & \tau_{\theta }^{2} \end{array}\right) \end{array}$$

As before, the within-study variances $\left(\text{V}(\left({\hat{\theta }}_{i}+{\hat{\gamma }}_{Wi}\right)\;\text{and}\;\text{V}\left({\hat{\theta }}_{i}\right)\right)$ are assumed known. Generally, the within-study correlation (*ρ*_*wi*(1,2)_) will be close to zero; males and females are different sets of individuals, but their treatment effects share a common adjustment for baseline values in model (8), which induces some correlation. Estimation of model (10) provides the summary treatment effect for males $\left({\hat{\mu }}_{\delta }\right)$, the summary treatment effect for females $\left({\hat{\mu }}_{\theta }\right)$, and the between-study variances (${\hat{\tau }}_{\gamma }^{2}$ and ${\hat{\tau }}_{\theta }^{2}$) and between-study correlation $\left({\hat{\rho }}_{B\left(1,2\right)}\right)$. The between-study correlation arises as studies with a larger true treatment effect for males may be associated with a larger (or smaller) treatment effect for females.

After fitting model (10), the summary interaction (difference in treatment effect for males and females) can be estimated by ${\hat{\mu }}_{\gamma }={\hat{\mu }}_{\delta }-{\hat{\mu }}_{\theta }$, with its $\text{variance}=\text{var}\left({\hat{\mu }}_{\delta }\right)+\mathrm{var}\left({\hat{\mu }}_{\theta }\right)-2\text{cov}\left({\hat{\mu }}_{\delta },{\hat{\mu }}_{\theta }\right)$. However, ${\hat{\mu }}_{\gamma }$ is no longer purely based on within-trial information (and hence why we have removed the ‘W’ term here) and rather is an amalgamation of the within-trial interactions (e.g. difference in treatment response between males and females in the same trial) and between-trial interaction (e.g. difference in mean treatment effect from a study with all males compared with a study with all females) (Riley *et al*., 2008b; Riley and Steyerberg, 2010; Thompson *et al*., 2010). To understand this, it is helpful to consider a study that only provides the treatment effect for females. This study can be included within the bivariate model (10) and would impact upon ${\hat{\mu }}_{\theta }$ and therefore also upon ${\hat{\mu }}_{\gamma }={\hat{\mu }}_{\delta }-{\hat{\mu }}_{\theta }$. Thus, the summary interaction estimate is influenced by a study that provides no estimate of within-trial interaction.

It can be shown that ${\hat{\mu }}_{\gamma }$ will be based solely on the between-trial interaction when trials only contain either males or females (i.e. when the within-trial interactions are not observable). Conversely, ${\hat{\mu }}_{\gamma }$ will be based solely on within-trial information when the proportion of males is the same in each trial (i.e. when between-trial interaction is not observable) (Simmonds and Higgins, 2007; Simmonds, 2005). Most IPD meta-analysis situations will be somewhere in between.

Some authors (e.g. (Riley *et al*., 2008b; Riley and Steyerberg, 2010; Thompson *et al*., 2010; Sutton *et al*., 2008; Jackson *et al*., 2006)) propose using ${\hat{\mu }}_{\gamma }$ (and thus combining within-trial and across-trial interactions) to gain power, especially when not all trials provide IPD. The use of ${\hat{\mu }}_{\gamma }$ is particularly appealing when the between-trial information has large precision (Simmonds and Higgins, 2007), as a result of large variation in covariate means across trials (an extreme example is when some trials contain only females and other trials only males). However, this approach implicitly assumes that the between-trial and within-trial interactions are the same. This is a strong assumption, as it is known that between-trial relationships are more prone to confounding and ecological bias. Many examples of this problem have been shown (Riley *et al*., 2008b; Riley and Steyerberg, 2010; Simmonds, 2005; Berlin *et al*., 2002).

For continuous covariates, model (10) is not appropriate as there are no longer two subgroups. However, an alternative is a bivariate meta-analysis with ${\hat{\theta }}_{i}$ (the treatment effect estimate for that with a zero covariate value) and ${\hat{\gamma }}_{Wi}$ (the change in treatment effect for a one-unit increase in the covariate), the two responses of interest. By accounting for within-study and between-study correlations, estimation of the bivariate model will give a summary interaction estimate that again combines the within-trial and between-trial interactions (Riley *et al*., 2008b; Riley and Steyerberg, 2010).

#### 6.1.1 Application to the hypertension data

Model (8) was applied to each trial in the hypertension data, with sex as a covariate and SBP as the outcome of interest. This produces the within-trial interaction estimate $\left({\hat{\gamma }}_{Wi}\right)$ and the treatment effect estimate for females $\left({\hat{\theta }}_{i}\right)$ for each trial, their variances $\left(V({\hat{\gamma }}_{Wi}\right)$and $V({\hat{\theta }}_{i}))$, and within-study correlations (*ρ*_*Wi*(1,2)_), which are close to zero. REML estimation of model (9) gave a summary within-trial interaction of ${\hat{\mu }}_{\gamma_{W}}=0.93$ (95% CI: −0.32 to 2.17; ${\hat{\tau }}_{\theta }=1.23$)). Thus, there is no evidence of an overall difference in treatment effect between males and females.

REML estimation of the bivariate model (10) gave a high summary treatment effect for females (${\hat{\mu }}_{\theta }=-10.66$; 95% CI: −12.76 to −8.55; ${\hat{\tau }}_{\theta }=2.94$)) and a high summary treatment effect for males (${\hat{\mu }}_{\delta }=-9.37$; 95% CI: −11.11 to −7.63)). The estimated between-study correlation was large (*ρ*_*B*_ = 0.86), indicating that populations with a larger treatment effect for males also have a larger treatment effect for females.

The summary interaction estimate from model (10) is ${\hat{\mu }}_{\gamma }={\hat{\mu }}_{\delta }-{\hat{\mu }}_{\theta }=1.28$ (95% CI: −0.23 to 2.80). This summary interaction combines the within-trial and between-trial interactions. It is noticeably larger than that based solely on within-trial information (1.28 vs 0.93) and has a wider CI as a result of larger estimates of the between-study variances in the bivariate model. Thus, the use of between-trial information is providing additional information here, although it may also be inducing ecological bias and study-level confounding. Nevertheless, all summary interaction estimates provide no evidence of a difference in treatment effect for males compared with females.

### 6.2 Prognostic or risk factor studies: combining partially and fully adjusted results

A major advantage of IPD is the ability to adjust for confounders and prognostic variables. This allows adjustment for baseline imbalance in randomised trials and can improve statistical power. In prognostic (or risk) factor studies, it also allows adjusted prognostic associations to be estimated, to reveal whether a novel factor retains its prognostic value after adjusting for existing prognostic factors. Nevertheless, even with IPD, some studies may not provide the adjustment variables of interest. In this setting, The Fibrinogen Studies Collaboration (The Fibrinogen Studies Collaboration, 2009) used a bivariate meta-analysis to account for the correlation between *partially* (P) adjusted and *fully* (F) adjusted prognostic factor effects, to borrow strength from partially adjusted effect estimates $\left({\hat{\theta }}_{iP}\right)$in studies where fully adjusted effect estimates $\left({\hat{\theta }}_{iF}\right)$ are unavailable. The model is as follows, and for full details, refer to The Fibrinogen Studies Collaboration, 2009:

(11) $$\begin{array}{cc} \left(\begin{array}{c} {\hat{\theta }}_{iP}\\ {\hat{\theta }}_{iF} \end{array}\right)\sim N\;\left(\left(\begin{array}{c} \theta_{iP}\\ \theta_{iF} \end{array}\right),\;\delta_{i}\right) & \delta_{i}=\left(\begin{array}{cc} S_{iP}^{2} & \rho_{Wi(P,F)}S_{iP}S_{iF}\\ \rho_{Wi(P,F)}S_{iP}S_{iF} & S_{iF}^{2} \end{array}\right)\\ \left(\begin{array}{c} \theta_{iP}\\ \theta_{iF} \end{array}\right)\sim N\;\left(\left(\begin{array}{c} \mu_{P}\\ \mu_{F} \end{array}\right),\;\Omega \right) & \Omega =\left(\begin{array}{cc} \tau_{P}^{2} & \tau_{P}\tau_{F}\rho_{B}\\ \tau_{P}\tau_{F}\rho_{B} & \tau_{F}^{2} \end{array}\right) \end{array}$$

The *s*_*iP*_ and *s*_*iF*_ are the standard errors of ${\hat{\theta }}_{iP}$ and ${\hat{\theta }}_{iF}$, respectively. Bootstrapping can estimate the within-study correlations (*ρ*_*Wi*(*P*,*F*)_) between partially and fully adjusted results in those studies that provide both (The Fibrinogen Studies Collaboration, 2009), as described in the preceding texts and shown in Web Appendix 5. A similar idea to utilise correlations to borrow strength is considered by others (Greenland, 1987; Steyerberg *et al*., 2000; Debray *et al*., 2012).

#### 6.2.1 Application to the hypertension data

As an illustration, assume that the first five trials in Table 1 provide IPD with treatment group, smoking, age and BMI available for all patients; however, the last five trials only provide IPD with smoking and treatment group. We now ask a new question: *Is smoking a prognostic factor for stroke*? Thus, the trials are now observational studies for this purpose. A Cox regression model can be fitted to the IPD for each of the 10 trials, to obtain partially adjusted hazard ratio estimates (that is unadjusted for age and BMI, but adjusted for treatment). In the first five trials, an extended Cox model can also be fitted to the IPD to obtain fully adjusted hazard ratio estimates (adjusted for age, BMI and treatment). Therefore, the first five trials provide both ${\hat{\theta }}_{iP}$ and ${\hat{\theta }}_{iF}$, whilst the last five trials only provide ${\hat{\theta }}_{iP}$. In the five trials with both, bootstrapping provides the within-study correlations for the partially and fully adjusted log hazard ratio estimates; these ranged from 0.89 to 0.99. Applying bivariate model (11) to all 10 trials using REML estimation gives a summary fully adjusted hazard ratio for smoking of 1.92 (95% CI: 1.44 to 2.57; ${\hat{\tau }}_{F}=0.18$); however, a univariate meta-analysis of just the five trials providing fully adjusted results gives a far higher summary hazard ratio of 2.70 (95% CI: 1.87 to 3.90; ${\hat{\tau }}_{P}\leq 0.001$). The utilisation of correlation thus reveals a lower adjusted hazard ratio than suggested by the five trials alone and a narrower 95% CI.

### 6.3 Longitudinal data

Jones *et al*. (2009) describe how to undertake a multivariate meta-analysis of randomised trials with longitudinal continuous outcomes. These essentially extend the models introduced in section 4, where the multiple outcomes now become multiple time points. The approach depends on whether one decides to model time as a factor or as continuous. When time is to be treated as a factor, the first step of the multivariate approach obtains the treatment effect estimates at each time point, with their variances and within-study correlations. When time is continuous with a linear trend assumed, differences in intercepts and slopes of the regression lines for the treatment and control groups are estimated, again with their variances and within-study correlation. The second step is then a multivariate meta-analysis of the trial estimates. Jones *et al*. (2009) show that accounting for the within-study correlations is important, as the estimate and standard error of the meta-analysis result at each time point can dramatically differ when naively assuming correlations are zero. However, they note that IPD studies are unlikely to provide repeated measurements at all the same follow-up times, and therefore, if time is analysed as a factor, similar time points (e.g. 6 months in trial 1 and 7 months in trial 2) may need to be grouped in order to proceed. Trikalinos and Olkin consider multivariate meta-analysis of treatment effect estimates at multiple time points for binary outcomes (Trikalinos and Olkin, 2012), and their formulae for deriving within-study correlations are easily applied with IPD.

### 6.4 Development of multiparameter models for prognostic, dose–response and diagnostic research

Another area where the whole regression equation (including the intercept or baseline hazard, if modelled) is important is for prognostic models (risk prediction models) (Steyerberg *et al*., 2013), where the fitted equation (e.g. logistic regression model) is needed to predict outcome risk for individuals based on their covariate values. Given IPD from multiple studies, the same prognostic model could be fitted in each study and their regression coefficients combined in a multivariate meta-analysis, to produce an overall model. This also allows the heterogeneity of effects for particular prognostic factors to be quantified and may guide decisions about which factors to include or exclude. For example, a factor with opposing effects across studies (e.g. OR > 1 in some studies, OR < 1 in others) may be best excluded, as a model will be more reliable for practice if the direction of a factor’s effect is consistent. If a factor has a consistent direction of effect but with varying magnitude (e.g. OR of 2 in some studies but 3 in other studies), then its inclusion may be warranted to improve a model’s discrimination, although potentially at the expense of reduced calibration (Debray *et al*., 2013).

Other uses of multivariate meta-analysis for multiparameter models are considered in detail elsewhere (Gasparrini and Armstrong, 2011; Gasparrini *et al*., 2012). One particularly important application is for examining dose–response relationships (Sauerbrei and Royston, 2011), for example, between the amount of alcohol intake and risk of cancer (Greenland and Longnecker, 1992; Orsini *et al*., 2012). Interest is in how increasing the dose or value of a factor increases (or decreases) the risk of a poor outcome. Linear and non-linear relationships can be fitted in each study, and then, a multivariate meta-analysis used to synthesise intercepts and slopes (for linearity) and additionally other terms (such as spline terms, quadratic terms, etc.) that allow for non-linearity, whilst accounting for the within-study correlation between all parameters. This produces a summary relationship across studies, and misleading inferences are possible if correlation is ignored. For example, Orsini *et al*. (Orsini *et al*., 2012) identify a linear trend between alcohol intake and lung cancer risk when using the multivariate approach (*p* = 0.03) but not when wrongly ignoring correlation (*p* = 0.58).

In diagnostic or screening test research, multivariate meta-analysis methods are being used to deal with correlation between sensitivity and specificity. Typically, such methods have allowed one pair of sensitivity and specificity estimates per study (Harbord *et al*., 2007). However, multiparameter models with multivariate meta-analysis can accommodate multiple pairs of results per study, which arise when test accuracy estimates are reported at multiple thresholds (Hamza *et al*., 2009; Riley *et al*., 2014). Monotonic relationships can be enforced within and between studies, such that sensitivity decreases and specificity increases as the threshold increases, and this enables a summary receiver operating characteristic curve to be produced.

### 6.5 Network meta-analysis

We have focused on multiple outcomes, but multivariate meta-analysis is also a natural framework for network meta-analysis of multiple treatments (White *et al*., 2012). Network meta-analysis relies heavily on comparability of different treatment comparisons (Salanti, 2012). IPD allows the analysis to be performed in an identical way in all studies – using the same outcome definition, the same analysis model and the same methods for covariate adjustment – and so should reduce the potential for inconsistency. Another possible source of inconsistency is treatment-by-covariate interaction, and IPD – even if only in a subset of trials – allows models to be fitted that allow for this interaction (Saramago *et al*., 2012; Donegan *et al*., 2013).

Recent work has included multiple outcomes within the network meta-analysis (Ades *et al*., 2010; Schmid *et al*., 2014; Efthimiou *et al*., 2014a; Efthimiou *et al*., 2014b) and includes an example where the ranking of treatments changes when the correlation between the multiple outcomes are accounted for (Efthimiou *et al*., 2014b). Within-study correlations are required when multiple outcomes are analysed or when multiarm trials are included (White *et al*., 2012); they can be estimated by the methods described in the preceding texts.

## 7 Discussion

We have described methods and applications of multivariate meta-analysis when IPD are available to calculate the within-study correlations, which are otherwise often difficult to calculate (Wei and Higgins, 2013b). Non-parametric bootstrapping is the most generally applicable method to estimate within-study correlations, although section 4 also described how to estimate them directly from a model for multiple continuous outcomes. By using correlations, the multivariate approach offers many potential benefits over separate univariate analyses. In our examples, we showed how multivariate meta-analysis allows joint probability inferences across outcomes, borrowing of strength (especially when some outcomes are (selectively) missing), more appropriate standard errors (e.g. for longitudinal data), and novel options to estimate adjusted results and treatment–covariate interactions. Many other novel applications are possible. For example, meta-analysis of surrogate outcome studies (Buyse *et al*., 2000; Michiels *et al*., 2009; Daniels and Hughes, 1997; Gail *et al*., 2000; Buyse, 2009), where a primary aim is to estimate and use the correlation between a surrogate outcome and a true outcome (such as between CD4 count and AIDS); prognostic studies where the absolute survival risk at multiple time points is needed (Arends *et al*., 2008); and investigations of the association between treatment effect and baseline risk (Van Houwelingen *et al*., 2002; Senn, 2010; Van Houwelingen *et al*., 1993).

We assumed that IPD are available in all studies. However, many of the methods and applications discussed in this paper allow the incorporation of additional non-IPD studies, if they directly provide estimates of (or data informing) some of the effects for the outcomes of interest. For example, adjusted prognostic factor results are notoriously prone to selective reporting bias, with non-significant adjusted results commonly excluded and only unadjusted or partially adjusted results shown. The bivariate model (11) allows non-IPD studies that provide only partially adjusted (or unadjusted) results to be included, and these may then still contribute (through the between-study correlation and any within-study correlations in IPD studies) towards the pooled adjusted result. One might expect the pooled adjusted estimate to be lower after accounting for studies that do not report adjusted results, as a result of their selective reporting. Even when non-IPD studies provide all the effects of interest, they are unlikely to provide their within-study correlations. Bujkiewicz *et al*., 2013 suggest including these studies by placing an informative prior distribution for their missing within-study correlations, based on the within-study correlation in available IPD studies. Specifically, they apply a double bootstrap procedure in an IPD study to estimate the within-study correlation and its uncertainty, which are then used to specify a prior distribution for the missing within-study correlations in other studies.

Some of the approaches discussed could also be fitted using a one-stage IPD model, in particular the continuous outcome models in section 4 (Riley *et al*., 2008b), the longitudinal modelling in section 6.4 (Jones *et al*., 2009), and the multiparameter modelling in section 6.5 (Orsini *et al*., 2012). This may be quicker and advantageous with rare events or small patient numbers, for example, to allow for more exact binomial or multinomial sampling distributions to be modelled within studies (Trikalinos *et al*., 2013; Trikalinos *et al*., 2014; Stijnen *et al*., 2010; Hamza *et al*., 2009). One-stage analysis may also be beneficial for advanced modelling of competing risks and correlated survival outcomes (Rondeau *et al*., 2011) and also multistate models (Price *et al*., 2011; Ades *et al*., 2010), which we have not considered here. However, one-stage models can be more computationally intensive, are difficult to specify for survival or mixed outcomes, and can have convergence issues (Riley *et al*., 2007b), which – in our experience – is particularly problematic when some outcomes are missing across studies. Both one-stage and two-stage models allow non-IPD studies to be combined with IPD studies where relevant (Riley *et al*., 2008b; Riley and Steyerberg, 2010; Sutton *et al*., 2008; Donegan *et al*., 2013; Riley *et al*., 2007c).

Finally, we recognise that the multivariate model is not without limitations (Jackson *et al*., 2011), even when IPD are available. In particular, there can be difficulty estimating between-study correlations (Riley *et al*., 2007b), and the use of a trial-level multivariate normal distribution assumes a linear relationship in the true effects across trials, which is hard to validate. In both one-stage and two-stage frameworks, the multivariate normal distribution is commonly assumed for the true effects across studies because it is convenient, can be implemented in most statistical software packages through REML estimation, and naturally extends the univariate setting where a normal distribution is often used. However, Baker and Jackson, 2008 note that, unlike within studies where the central limit theorem suggests that normal sampling distributions of effect estimates will occur when sample sizes become large, between studies “the Central Limit Theorem does not really imply anything for the distribution of the random effect ….We can only appeal to the Central Limit Theorem here with the vague idea that the unknown source of variation between studies might be the sum of several factors”. If one wishes to avoid the multivariate normality assumption between studies, then non-parametric estimation methods can be used such as MM (Jackson *et al*., 2010; Jackson *et al*., 2013; Chen *et al*., 2012; DerSimonian and Laird, 1986). However, if interested in the joint predictive distribution of effects, then a parametric specification is preferable. Lee and Thompson, 2008 suggest a number of flexible alternatives to the normality assumption, for both univariate and bivariate settings, whilst a longer tailed distribution has been proposed in the univariate setting (Baker and Jackson, 2008). Recently, in the setting of test accuracy studies where sensitivity and specificity are of interest, a one-stage meta-analysis using a beta-binomial model has been proposed (Kuss *et al*., 2014), with the correlation between sensitivity and specificity accounted for using bivariate copulas.

In conclusion, with its potential statistical benefits and a wide range of areas for application, multivariate meta-analysis is likely to gain increasing interest in the future, and we have shown how to implement the approach when IPD are available from multiple studies.

## Supporting information

Additional supporting information may be found in the online version of this article at the publisher’s web site.

Supplementary Material

## Figures and Tables

**Table 1.** Summary results for the 10 trials included in the meta-analysis of Wang *et al*. (Wang *et al*., 2005)

								Within-study correlations						
		Number of patients		ANCOVA^b^ treatment effect outcomes 1 and 2		Cox regression treatment effect outcomes 3 and 4		SBP and DBP	SBP and DBP	SBP and CVD	SBP and stroke	DBP and CVD	DBP and stroke	CVD and stroke
					
				SBP	DBP	CVD	Stroke							
ID (*i*)	Trial name^a^	Control	Treatment	Mean difference (var)	Mean difference (var)	log(HR) (var)	log(HR) (var)	From model (3)	From bootstrap	From bootstrap	From bootstrap	From bootstrap	From bootstrap	From bootstrap
1	ATMH	750	780	−6.66 (0.72)	−2.99 (0.27)	−0.09 (0.17)	−1.91 (1.17)	0.78	0.79	0.01	−0.01	−0.02	−0.02	0.16
2	HEP	199	150	−14.17 (4.73)	−7.87 (1.44)	0.06 (0.13)	−0.15 (0.17)	0.45	0.50	0.11	0.10	0.09	0.10	0.64
3	EWPHE	82	90	−12.88 (10.31)	−6.01 (1.77)	−0.17 (0.20)	0.75 (0.35)	0.59	0.59	−0.21	−0.05	−0.04	−0.04	0.10
4	HDFP	2371	2427	−8.71 (0.30)	−5.11 (0.10)	−0.24 (0.03)	−0.29 (0.07)	0.77	0.77	0.09	0.02	0.13	0.04	0.52
5	MRC-1	3445	3546	−8.70 (0.14)	−4.64 (0.05)	−0.18 (0.03)	−0.41 (0.11)	0.66	0.64	0.04	0.04	0.04	0.04	0.42
6	MRC-2	1337	1314	−10.60 (0.58)	−5.56 (0.18)	−0.23 (0.02)	−0.20 (0.03)	0.49	0.50	0.00	0.03	−0.02	0.00	0.62
7	SHEP	2371	2365	−11.36 (0.30)	−3.98 (0.075)	−0.32 (0.02)	−0.45 (0.02)	0.50	0.48	−0.01	−0.02	−0.03	−0.03	0.69
8	STOP	131	137	−17.93 (5.82)	−6.54 (1.31)	−1.87 (1.17)	0.32 (0.83)	0.61	0.59	−0.02	−0.07	−0.03	0.00	0.35
9	Sy-Chi	1139	1252	−6.55 (0.41)	−2.08 (0.11)	−0.33 (0.09)	−0.48 (0.04)	0.45	0.45	0.11	0.08	0.03	0.03	0.78
10	Sy-Eur	2297	2398	−10.26 (0.20)	−3.49 (0.04)	−0.26 (0.03)	−0.55 (0.03)	0.51	0.48	0.05	0.04	0.04	0.05	0.62

a Trial names are consistent with Wang *et al*. (Wang *et al*., 2005), where further details and trial publications can be found.

b Treatment effect is the mean difference (treatment group minus control group) in the patients’ final blood pressure (follow-up minus baseline) after adjusting for baseline values; these data correct those erroneously displayed elsewhere (Riley *et al*., 2008b; Jackson *et al*., 2013).

SBP, systolic blood pressure; DBP, diastolic blood pressure; sd, standard deviation; var, variance; CVD, cardiovascular disease.

**Table 2.** Bivariate and multivariate meta-analysis results for the hypertension data as obtained from REML estimation

Model	Outcome	Effect type	Summary treatment effect	95% CI for the summary effect^^^	Between-study standard deviation(s)*	Between-study correlation(s)
Bivariate^a^	SBP	Mean difference	−10.21	−12.11 to −8.30	2.71	SBP, DBP 0.78
	DBP	Mean difference	−4.59	−5.61 to −3.57	1.48	
Bivariate	SBP ≤ 120	Odds ratio	2.45	1.96 to 3.06	0.119	SBP, DBP 0.35
	DBP ≤ 80	Odds ratio	2.34	2.01 to 2.72	0.204	
Bivariate	CVD	HR	0.78	0.69 to 0.89	<0.000001	CVD, stroke 1.00**
	Stroke	HR	0.68	0.60 to 0.78	<0.000001	
Multivariate	SBP	Mean difference	−10.22	−12.14 to −8.30	2.73	SBP, DBP 0.79
	DBP	Mean difference	−4.63	−5.67 to −3.60	1.51	SBP, CVD −0.31
	CVD	HR	0.79	0.69 to 0.91	0.05	SBP, stroke −0.53
	Stroke	HR	0.73	0.61 to 0.87	0.14	DBP, CVD −0.83
						DBP, stroke −0.94
						CVD, stroke 0.97

a These results correct those bivariate REML results displayed elsewhere that used slightly different trial estimates and variances for this hypertension data (Riley *et al*., 2008b; Jackson *et al*., 2013).

^ Inflated to account for uncertainty in the estimation of between-study standard deviation and correlation.

* Measured on the log HR scale for stroke and CVD and on log OR scale for SBP ≤ 120 and DBP ≤ 80.

** Estimation at edge of boundary space is a consequence of between-study standard deviation estimates close to zero.
