# Supplementary material for: Multivariate meta-analysis using individual participant data
Source: Res Synth Methods. Author manuscript; Available in PMC 2016 Apr 27. (PMC4847645; doi:10.1002/jrsm.1129)
Supplement: Supplementary Material [file NIHMS67641-supplement-Supplementary_Material.docx]

**Web Appendix 1**: WinBUGS code to fit a Bayesian multivariate meta-analysis of 4 outcomes, using either Cholesky or spherical decomposition

**model {**

# Loop over number of studies

for (n in 1:N) {

# Within study plus between study covariance matrix, and then their inverse

covariance[n,1,1] <- varianceY1[n] + tau[1]

covariance[n,2,2] <- varianceY2[n] + tau[2]

covariance[n,3,3] <- varianceY3[n] + tau[3]

covariance[n,4,4] <- varianceY4[n] + tau[4]

covariance[n,1,2] <- cov12[n] + (rhob12 * sqrt(tau[1] * tau[2]))

covariance[n,2,1] <- covariance[n,1,2]

covariance[n,1,3] <- cov13[n] + (rhob13 * sqrt(tau[1] * tau[3]))

covariance[n,3,1] <- covariance[n,1,3]

covariance[n,1,4] <- cov14[n] + (rhob14 * sqrt(tau[1] * tau[4]))

covariance[n,4,1] <- covariance[n,1,4]

covariance[n,2,3] <- cov23[n] + (rhob23 * sqrt(tau[2] * tau[3]))

covariance[n,3,2] <- covariance[n,2,3]

covariance[n,2,4] <- cov24[n] + (rhob24 * sqrt(tau[2] * tau[4]))

covariance[n,4,2] <- covariance[n,2,4]

covariance[n,3,4] <- cov34[n] + (rhob34 * sqrt(tau[3] * tau[4]))

covariance[n,4,3] <- covariance[n,3,4]

prec[n,1:4,1:4] <- inverse(covariance[n, , ])

# Likelihood

Y[n,1:4] ~ dmnorm(mu[1:4],prec[n,1:4,1:4])

}

# Variance Priors 1

tau[1] <- sd[1] * sd[1]

tau[2] <- sd[2] * sd[2]

tau[3] <- sd[3] * sd[3]

tau[4] <- sd[4] * sd[4]

sd[1] ~ dnorm(0,1)I(0,)

sd[2] ~ dnorm(0,1)I(0,)

sd[3] ~ dnorm(0,1)I(0,)

sd[4] ~ dnorm(0,1)I(0,)

# Correlation matrix (R) priors using Decomposition

rhob12 <- L[1,1] * L[1,2]

rhob13 <- L[1,1] * L[1,3]

rhob14 <- L[1,1] * L[1,4]

rhob23 <- L[1,2] * L[1,3] + L[2,2] * L[2,3]

rhob24 <- L[1,2] * L[1,4] + L[2,2] * L[2,4]

rhob34 <- L[1,3] * L[1,4] + L[2,3] * L[2,4] + L[3,3] * L[3,4]

# 1. Spherical Decomposition - equation (7) Wei and Higgins

L[1,1] <- 1

L[1,2] <- cos(phi[2,1])

L[1,3] <- cos(phi[3,1])

L[1,4] <- cos(phi[4,1])

L[2,2] <- sin(phi[2,1])

L[2,3] <- sin(phi[3,1]) * cos(phi[3,2])

L[2,4] <- sin(phi[4,1]) * cos(phi[4,2])

L[3,3] <- sin(phi[3,1]) * sin(phi[3,2])

L[3,4] <- sin(phi[4,1]) * sin(phi[4,2]) * cos(phi[4,3])

L[4,4] <- sin(phi[4,1]) * sin(phi[4,2]) * sin(phi[4,3])

phi[2,1] ~ dunif(0,3.1415)

phi[3,1] ~ dunif(0,3.1415)

phi[3,2] ~ dunif(0,3.1415)

phi[4,1] ~ dunif(0,3.1415)

phi[4,2] ~ dunif(0,3.1415)

phi[4,3] ~ dunif(0,3.1415)

# 2. Cholesky Decomposition - equation (5) Wei and Higgins

#L[1,1] <- 1

#L[1,2] ~ dunif(-1,1)

#L[1,3] ~ dunif(-1,1)

#L[1,4] ~ dunif(-1,1)

#L[2,2] <- sqrt(1 - pow(L[1,2],2))

#L.u[2,3] ~ dunif(-1,1)

#L.r[2,3] <- - sqrt(1 - pow(L[1,3],2))

#L[2,3] <- L.u[2,3] * L.r[2,3]

#L.u[2,4] ~ dunif(-1,1)

#L.r[2,4] <- - sqrt(1 - pow(L[1,4],2))

#L[2,4] <- L.u[2,4] * L.r[2,4]

#L[3,3] <- sqrt(1 - pow(L[1,3],2) - pow(L[2,3],2))

#L.u[3,4] ~ dunif(-1,1)

#L.r[3,4] <- sqrt(1 - pow(L[1,4],2) - pow(L[2,4],2))

#L[3,4] <- L.u[3,4] * L.r[3,4]

#L[4,4] <- sqrt(1 - pow(L[1,4],2) - pow(L[2,4],2) -

# pow(L[3,4],2))

# Between study Covariance matrix

pred.covmat[1,1] <- tau[1]

pred.covmat[2,2] <- tau[2]

pred.covmat[3,3] <- tau[3]

pred.covmat[4,4] <- tau[4]

pred.covmat[1,2] <- rhob12 * sqrt(tau[1] * tau[2])

pred.covmat[2,1] <- rhob12 * sqrt(tau[1] * tau[2])

pred.covmat[1,3] <- rhob13 * sqrt(tau[1] * tau[3])

pred.covmat[3,1] <- rhob13 * sqrt(tau[1] * tau[3])

pred.covmat[1,4] <- rhob14 * sqrt(tau[1] * tau[4])

pred.covmat[4,1] <- rhob14 * sqrt(tau[1] * tau[4])

pred.covmat[2,3] <- rhob23 * sqrt(tau[2] * tau[3])

pred.covmat[3,2] <- rhob23 * sqrt(tau[2] * tau[3])

pred.covmat[2,4] <- rhob24 * sqrt(tau[2] * tau[4])

pred.covmat[4,2] <- rhob24 * sqrt(tau[2] * tau[4])

pred.covmat[3,4] <- rhob34 * sqrt(tau[3] * tau[4])

pred.covmat[4,3] <- rhob34 * sqrt(tau[3] * tau[4])

pred.prec[1:4,1:4] <- inverse(pred.covmat[ , ])

# Mean Priors 1

mu[1] ~ dnorm(0.0,1.0E-6)

mu[2] ~ dnorm(0.0,1.0E-6)

mu[3] ~ dnorm(0.0,1.0E-6)

mu[4] ~ dnorm(0.0,1.0E-6)

# Results

# Transformed mean

expmu[1] <- exp(mu[1])

expmu[2] <- exp(mu[2])

expmu[3] <- exp(mu[3])

expmu[4] <- exp(mu[4])

# Predictive distribution

pred[1:4] ~ dmnorm(mu[1:4],pred.prec[1:4,1:4])

exppred[1] <- exp(pred[1])

exppred[2] <- exp(pred[2])

exppred[3] <- exp(pred[3])

exppred[4] <- exp(pred[4])

# prob reduces SBP and DBP by 5 points or more vs placebo

# in a new study

#SBP.ind <- 1 - equals(min(pred[1],-5),-5)

#DBP.ind <- 1 - equals(min(pred[2],-5),-5)

#both.ind <- SBP.ind * DBP.ind

# prob reduces SBP and DBP by >0 points or more vs placebo

# in a new study

#SBP.ind <- 1 - equals(min(pred[1],0),0)

#DBP.ind <- 1 - equals(min(pred[2],0),0)

#both.ind <- SBP.ind * DBP.ind

# prob reduces SBP and DBP by 5 points or more vs placebo

# ON AVERAGE

#mean.SBP.ind <- 1 - equals(min(mu[1],-5),-5)

#mean.DBP.ind <- 1 - equals(min(mu[2],-5),-5)

#mean.both.ind <- mean.SBP.ind * mean.DBP.ind

# prob reduces SBP and DBP by >0 points or more vs placebo

# ON AVERAGE

#mean.SBP.ind <- 1 - equals(min(mu[1],0),0)

#mean.DBP.ind <- 1 - equals(min(mu[2],0),0)

#mean.both.ind <- mean.SBP.ind * mean.DBP.ind

# prob reduces SBP by 10 and stroke by 20% vs placebo

# in a new study

SBP.ind <- 1 - equals(min(pred[1],-5),-5)

stroke.ind <- 1 - equals(min(exppred[4],0.8),0.8)

both.ind <- SBP.ind * stroke.ind

**}**

# Data - blood pressure outcomes (Y1 SBP and Y2 DBP) and

# survival outcomes (CVD Y3 and stroke Y4)

list(

N=10,

varianceY1=c(

0.7230616, 4.728005, 10.30973, 0.2951848, 0.1405789,

0.5842536, 0.2984707, 5.821138, 0.4144522, 0.1952633

),

varianceY2=c(

0.2722278, 1.435314, 1.770885, 0.1032037, 0.0504897,

0.1825371, 0.0745742, 1.311613, 0.1067053, 0.0424293

),

varianceY3=c(.1667308, .1256746, .2008801, .0291161, .0304781, .0241523, .0167078, 1.168684, .0935472, .0314769),

varianceY4=c(

1.167421, 0.171657, 0.3505942, 0.0656085, 0.1121221,

0.0318311, 0.0159989, 0.833896, 0.0400794, 0.0342735

),

cov12=c(.3504944, 1.302514, 2.520989, .1343957, .0539189, .163285, .0716121, 1.630268, .094633, .0436902),

cov13=c(.0034721, .0847921, -.3022118, .0083436, .0026183, 0, -.0007062, -.0521654, .0216593, .0039199),

cov14=c(-.0091876, .0900886, -.0950596, .0027833, .0050219, .0040912, -.0013821, -.1542261, .0103107, .0032723),

cov23=c(-.0042609, .0382243, -.0238574, .0071262, .0015691, -.001328, -.0010589, -.0371426, .0029973, .0014618),

cov24=c(-.0112748, .0496368, -.0315179, .0032915, .0030096, 0, -.0010362, 0, .0019619, .0019067),

cov34=c(.0705897, .0940014, .0265382, .0227274, .0245521, .0171908, .0112812, .3455194, .0477607, .0203642),

Y=structure(.Data=c(

-6.664233, -2.988614, -.0879826, -1.91358,

-14.16601, -7.874312, .0585179, -.1459925,

-12.88107, -6.011509, -.166865, .753264,

-8.708664, -5.109798, -.2440465, -.2875622,

-8.701553, -4.63645, -.1795313, -.4084455,

-10.60237, -5.564794, -.2285552, -.1985812,

-11.35744, -3.977884, -.3158144, -.4495284,

-17.92623, -6.537015, -1.873505, .3170643,

-6.548491, -2.084006, -.3260556, -.48373,

-10.25582, -3.494257, -.2566346, -.5534967

), .Dim=c(10,4))

)

# Initial values 1 - Cholesky

list(

sd=c(3,2,0.1,0.1),

mu = c(-5,-5,-0.5,-0.5),

pred=c(-5,-5,-0.5,-0.5),

L =structure(.Data=c(

NA,-0.99,-0.99,-0.99,

NA,NA,NA,NA,

NA,NA,NA,NA,

NA,NA,NA,NA

), .Dim=c(4,4)),

L.u =structure(.Data=c(

NA,NA,NA,NA,

NA,NA,-0.99,-0.99,

NA,NA,NA,-0.99

), .Dim=c(3,4))

**)**

# Initial values 2 - Cholesky

list(

sd=c(3,2,0.1,0.1),

mu = c(5,5,0.5,0.5),

pred=c(5,5,0.5,0.5),

L =structure(.Data=c(

NA,0.99,0.99,0.99,

NA,NA,NA,NA,

NA,NA,NA,NA,

NA,NA,NA,NA

), .Dim=c(4,4)),

L.u =structure(.Data=c(

NA,NA,NA,NA,

NA,NA,0.99,0.99,

NA,NA,NA,0.99

), .Dim=c(3,4))

**)**

# Initial values 1 - Spherical

list(

sd=c(3,2,0.1,0.1),

mu = c(-5,-5,-0.5,-0.5),

pred=c(-5,-5,-0.5,-0.5)#,

#phi =structure(.Data=c(

#NA,NA,NA,NA,

#0.01,NA,NA,NA,

#0.01,0.01,NA,NA,

#0.01,0.01,0.01,NA

#), .Dim=c(4,4))

**)**

# Initial values 2 - Spherical

list(

sd=c(3,2,0.1,0.1),

mu = c(-5,-5,-0.5,-0.5),

pred=c(-5,-5,-0.5,-0.5)#,

#phi =structure(.Data=c(

#NA,NA,NA,NA,

#3.1414,NA,NA,NA,

#3.1414,3.1414,NA,NA,

#3.1414,3.1414,3.1414,NA

#), .Dim=c(4,4))

**)**

**Web Appendix 2**: WinBUGS code to fit a Bayesian bivariate meta-analysis

**model {**

# Loop over number of studies

for (n in 1:N) {

# Within study plus between study covariance matrix, and then their inverse

covariance[n,1,1] <- varianceY1[n] + tau[1]

covariance[n,2,2] <- varianceY2[n] + tau[2]

covariance[n,1,2] <- cov[n] + (rhob * sqrt(tau[1] * tau[2]))

covariance[n,2,1] <- covariance[n,1,2]

prec[n,1:2,1:2] <- inverse(covariance[n, , ])

# Likelihood

Y[n,1:2] ~ dmnorm(mu[1:2],prec[n,1:2,1:2])

}

# Variance Priors 1

tau[1] <- sd[1] * sd[1]

tau[2] <- sd[2] * sd[2]

sd[1] ~ dnorm(0,1)I(0,)

sd[2] ~ dnorm(0,1)I(0,)

pred.covmat[1,1] <- tau[1]

pred.covmat[1,2] <- rhob * sqrt(tau[1] * tau[2])

pred.covmat[2,1] <- rhob * sqrt(tau[1] * tau[2])

pred.covmat[2,2] <- tau[2]

pred.prec[1:2,1:2] <- inverse(pred.covmat[ , ])

# Mean Priors 1

mu[1] ~ dnorm(0.0,1.0E-6)

mu[2] ~ dnorm(0.0,1.0E-6)

rhob ~ dunif(0,1)

# Results

# Transformed mean

expmu[1] <- exp(mu[1])

expmu[2] <- exp(mu[2])

# Predictive distribution

pred[1:2] ~ dmnorm(mu[1:2],pred.prec[1:2,1:2])

exppred[1] <- exp(pred[1])

exppred[2] <- exp(pred[2])

# prob reduces SBP and DBP by 5 points or more vs placebo

# in a new study

# include when using Data 1

SBP.ind <- 1 - equals(min(pred[1],-5),-5)

DBP.ind <- 1 - equals(min(pred[2],-5),-5)

both.ind <- SBP.ind * DBP.ind

# prob reduces SBP and DBP by >0 points or more vs placebo

# in a new study

#SBP.ind <- 1 - equals(min(pred[1],0),0)

#DBP.ind <- 1 - equals(min(pred[2],0),0)

#both.ind <- SBP.ind * DBP.ind

# prob reduces SBP and DBP by 5 points or more vs placebo

# ON AVERAGE

# include when using Data 1

mean.SBP.ind <- 1 - equals(min(mu[1],-5),-5)

mean.DBP.ind <- 1 - equals(min(mu[2],-5),-5)

mean.both.ind <- mean.SBP.ind * mean.DBP.ind

# prob reduces SBP and DBP by >0 points or more vs placebo

# ON AVERAGE

#mean.SBP.ind <- 1 - equals(min(mu[1],0),0)

#mean.DBP.ind <- 1 - equals(min(mu[2],0),0)

#mean.both.ind <- mean.SBP.ind * mean.DBP.ind

# Data - blood pressure outcomes (SBP and DBP)

list(

# Y1 = Systolic blood pressure

# Y2 = Diastolic blood pressure

N=10,

varianceY1=c(

0.7230616, 4.728005, 10.30973, 0.2951848, 0.1405789,

0.5842536, 0.2984707, 5.821138, 0.4144522, 0.1952633

),

varianceY2=c(

0.2722278, 1.435314, 1.770885, 0.1032037, 0.0504897,

0.1825371, 0.0745742, 1.311613, 0.1067053, 0.0424293

),

cov=c(.3504944, 1.302514, 2.520989, .1343957, .0539189, .163285, .0716121, 1.630268, .094633, .0436902),

Y=structure(.Data=c(

-6.664233, -2.988614,

-14.16601, -7.874312,

-12.88107, -6.011509,

-8.708664, -5.109798,

-8.701553, -4.63645,

-10.60237, -5.564794,

-11.35744, -3.977884,

-17.92623, -6.537015,

-6.548491, -2.084006,

-10.25582, -3.494257

), .Dim=c(10,2))

)

# Initial values

list(

sd=c(1,1),

rhob=0.5,

mu = c(-5,-5)

)

**Web Appendix 3**: SAS code for fitting model (3) to each trial separately

Model (3) can be rewritten using dummy variables, as follows:

**

where *I.SBP_k_* = 1 if the response is for outcome *k* = 1 and 0 otherwise, and *I.DBP_k_* = 1 if the response is for outcome *k* = 2 and 0 otherwise. This can then be specified in SAS as follows:

proc mixed cl method=reml data=hypertension;

/* 'idnr' is unique patient identification number, and 'trial' is unique trial identifier */

class idnr trial ;

by trial;

/* bpl is a single response column containing two records for each patient, one for their last

SBP and one for their last DBP; sbpi and dbpi are the initial SBP and DBP values respectively; sbp is a dummy variable, equal to 1 when the response relates to SBP and 0 otherwise; and dbp is a dummy variables equal to 1 when the response related to DBP and 0 otherwise; corrb provides the correlation matrix of the estimated effects, and covb provides the covariance matrix; noint ensures no additional intercept term is included */

model bpl = sbpi*sbp dbpi*dbp sbp dbp sbp*treat dbp*treat / noint s cl covb corrb residual ;

/* the repeated statement ensures a separate residual variance for each outcome, and allows for the correlation between the residuals of the two outcomes */

repeated / type = arh(1) subject = trial(idnr);

/*starting values for the residual variances */

parms

(0.1)

(0.1)

(0.01)

;

run;

**Web Appendix 4:** Multivariate meta-analysis data of the treatment effects for the two binary outcomes of a normal SBP $\leq$ 120 mmHg and a normal DBP $\leq$ 80 mmHg

| **SBP** | **DBP** | **SBP** | **DBP** | Within-study covariance |
| --- | --- | --- | --- | --- |
| logOR | logOR | Var(logOR) | Var(logOR) |  |
| 1.0929 | 0.59832 | 0.022536 | 0.021077 | 0.010418 |
| -0.4594 | 1.1704 | 1.516765 | 0.054864 | 0.015376 |
| -0.10223 | 0.93115 | 2.026611 | 0.121661 | 0.052733 |
| 0.8691 | 0.794803 | 0.004826 | 0.00536 | 0.002447 |
| 0.97788 | 0.810344 | 0.006597 | 0.004148 | 0.002018 |
| 0.852899 | 0.887123 | 0.077437 | 0.006878 | 0.003083 |
| 0.98 | 1.072312 | 0.019944 | 0.007584 | 0.001118 |
| -0.76866 | 1.118318 | 1.526823 | 0.097744 | 0.06915 |
| 0.269759 | 0.427979 | 0.066904 | 0.00745 | 0.003193 |
| 0.30515 | 1.012815 | 0.143034 | 0.004543 | 0.002223 |

**Web Appendix 5**: STATA code to obtain bootstrap estimates of the within-study correlation between each pair of the four outcomes

/* define program to run linear regressions for SBP and DBP and Cox models for CVD and stroke */

prog def myprog4, rclass

regress sbpl sbpi treat

return scalar sbptreat = _b[treat]

regress dbpl dbpi treat

return scalar dbptreat = _b[treat]

stset dl_cv, failure(cv==1)

stcox treat, nohr

return scalar cvtreat = _b[treat]

stset dl_st, failure(st==1)

stcox treat, nohr

return scalar stroketreat = _b[treat]

end

/* for each trial separately, obtain 2000 bootstrap estimates of the four treatment effects */

keep if trial == "ANBP"

bootstrap b_treat_sbp=r(sbptreat) b_treat_dbp=r(dbptreat) b_treat_cv=r(cvtreat) b_treat_stroke=r(stroketreat) , saving(boot, replace) rep(2000) seed(231): myprog4

/* estimate the within-study correlations */

use boot, clear

corr b_treat_sbp b_treat_dbp b_treat_cv b_treat_stroke
